# Increasing survival disparity between children, adolescents, and young adults with osteosarcoma or Ewing sarcoma of bone from 1990 to 2024: a population-based cohort study

**DOI:** 10.2340/ao.v65.45405

**Published:** 2026-04-22

**Authors:** Daniel Thor Halberg Dybdal, Klaus Rostgaard, Henrik Hjalgrim, Ninna Aggerholm-Pedersen, Niels Junker, Thomas Baad-Hansen, Pernille Wendtland Edslev, Eva Kristine Ruud Kjær, Akmal Safwat, Michael Mørk Petersen, Lisa Lyngsie Hjalgrim

**Affiliations:** a Department of Paediatrics and Adolescent Medicine, Copenhagen University Hospital Rigshospitalet, Copenhagen, Denmark; b Danish Cancer Institute, Danish Cancer Society, Copenhagen, Denmark; c Department of Clinical Medicine, University of Copenhagen, Copenhagen, Denmark; d Department of Haematology, Copenhagen Univerity Hospital Rigshospitalet, Copenhagen, Denmark; e Department of Oncology, Aarhus University Hospital, Aarhus, Denmark; f Department of Clinical Medicine, Aarhus University, Aarhus, Denmark; g Department of Oncology, Herlev and Gentofte Hospital, Copenhagen, Denmark; h Department of Orthopaedic Oncology, Aarhus University Hospital, Aarhus, Denmark; i Department of Paediatrics and Adolescent Medicine, Aarhus University Hospital, Aarhus, Denmark; j Danish Center for Particle Therapy, Aarhus University Hospital, Aarhus, Denmark; k Department of Orthopaedic Surgery, Copenhagen University Hospital Rigshospitalet, Copenhagen, Denmark

**Keywords:** Osteosarcoma, Ewing sarcoma, cohort study, incidence, mortality, child, adolescent, young adult

## Abstract

**Background and purpose:**

Bone sarcomas are important contributors to early-life cancer mortality and morbidity. Optimisation of treatment regimens has improved survival, but the survival disparity between children, adolescents and young adults has widened. Population-based studies are needed to understand and address these disparities. This is the first comprehensive study of early-life bone sarcomas in Denmark.

**Patient/material and methods:**

We combined population-wide data from national registers from 1990 to 2024 with clinical data from patient records. We calculated age-standardised incidence rates, 5-year relative survival rates stratified by several clinical factors, and Aalen-Johansen estimators for relapse/progression and death within 5 years.

**Results:**

A total of 578 patients under 40 years of age were diagnosed with either osteosarcoma (*n* = 336) or Ewing sarcoma of bone (*n* = 242) in Denmark between 1990 and 2024. Five-year relative survival improved for patients aged 0–24 years but stagnated for those aged 25–39 years. We observed age-dependent differences in the distribution of tumours and in relative survival across anatomical sites, tumour sizes, and treatment regimens. Metastatic disease or a tumour diameter of > 8 cm reduced relative survival by 19 to 45 percentage points.

**Interpretation:**

Survival disparities between children, adolescents, and young adults with bone sarcomas are likely multifactorial. Age-dependent differences in the distribution of tumours across anatomical sites and in tumour size appear to play a role. The incidence of and survival from relapse/progression also appear to favour younger patients. Including patients in international, joint paediatric-adult treatment protocols remains a high priority.

## Introduction

Bone sarcomas are the third-leading cause of cancer death in children, adolescents, and young adults (CAYA) in high-income settings [1]. Osteosarcoma and Ewing sarcoma are the two most common bone tumours in CAYA. Both diseases have incidence peaks spanning school-age, adolescence, and early adulthood, and both are highly malignant and require intensive, multimodal treatment. Historically, these bone cancers have had a poor prognosis, with abysmal survival rates for metastatic or relapsed disease. For decades, no new treatment modalities have been introduced. Instead, treatment protocols, driven by international, multidisciplinary consortia, have focused on improving stratification and optimising treatment courses using existing treatment options [2–8]. Increased use of such protocols has improved survival, primarily in patients with localised disease [9, 10]. However, these advances have not benefitted patients equally, with only marginal survival gains observed in older adolescents and young adults, leading to widening survival disparities [11–15].

In other early-life cancers, survival among young adult patients improved after the implementation of joint paediatric-adult treatment protocols [16, 17]. Over the past three decades, similar efforts have been made to integrate paediatric and adult patients with bone sarcoma into multinational, investigator-initiated clinical trials [2–8, 18]. Despite these efforts, our understanding of age-dependent survival disparities and how to address them remains limited.

Such disparities are likely influenced by national and regional factors, including differences in the implementation and recruitment to the aforementioned protocols, structural differences in healthcare services, and population-level socio-economic differences. Thus, while pooling multinational data is important for studying rare cancers, national population-based studies are equally necessary to improve patient outcomes in practice. There have been no previous comprehensive studies of Danish CAYA with primary bone sarcomas.

In this population-based study, we describe survival disparities among clinically relevant subgroups of CAYA diagnosed with osteosarcoma or Ewing sarcoma of bone in Denmark and identify potential contributing factors. To do so, we combine three decades of national register data with clinical data from individual patient records.

## Patients/material and methods

### Study population data

Using the Danish Cancer Register [19], we identified all individuals diagnosed with osteosarcoma or Ewing sarcoma of bone under the age of 40 years between 1990 and 2024. We first filtered by histological diagnosis using morphology codes from the International Classification of Diseases for Oncology, 3rd Edition (ICD-O-3) [20]. We included morphology codes corresponding to all osteosarcoma subtypes and Ewing sarcoma. To accommodate changes in diagnostic practices and the categorisation of Ewing-like tumours, we also included tumours coded as Askin tumours and peripheral primitive neuroectodermal tumours. We subsequently filtered on ICD-O-3 topology codes to include only bone tumours. The morphology and topology codes used, and their distribution within the study population, are shown in Supplementary Materials 1. Patients diagnosed in 2002–2024 were validated using the Danish Pathology Data Bank, which automatically registers all pathology examinations performed in Danish healthcare institutions [19]. We extracted records from 2002–2024 in which any bone sarcoma morphology code was used as either a confirmed or suspected diagnosis, and manually screened electronic hospital records to confirm or exclude a diagnosis of osteosarcoma or Ewing sarcoma in a bone.

There are no universally agreed age cut-offs separating childhood, adolescence, and young adulthood. The upper limit of young adulthood is also not clearly defined, but both the European Society for Medical Oncology and the US National Cancer Institute use an inclusive definition with an upper age limit of 39 years [21, 22]. We used two age-group categorisations to facilitate comparisons with previous studies that used varying age definitions. Our primary categorisation divided participants into three age groups (0–14, 15–24, and 25–39 years) to reflect biological and behavioural differences between CAYA. Our secondary categorisation used two groups (0–17 and 18–39 years), corresponding to the age ranges typically treated in paediatric versus adult oncology units.

From the Danish Civil Registration System [19], we extracted dates of birth, death, migration, or disappearance. The end of follow-up for vital status was December 31, 2025.

For patients diagnosed in 2002–2024, clinical data on disease stage, tumour location and size, treatment regimen, and progression/relapse were extracted manually from patients’ electronic health records. Progression during first-line treatment and/or relapse after were combined into a single variable. There is no consensus definition on what constitutes a small or large primary tumour for either sarcoma type. We used a dichotomous definition of tumour size based on the largest tumour diameter at diagnosis, using a cutoff of 8 cm, which was the most commonly used in previous studies of osteosarcoma and Ewing sarcoma [13, 23].

The study was additionally supplemented with data from The Danish Cancer Register, the Danish Sarcoma Database and the Danish Childhood Cancer Register, which are described in detail in other publications [19, 24, 25].

### Statistical analyses

All primary analyses were stratified by six main groups, defined by sarcoma type (osteosarcoma and Ewing sarcoma of bone) and three age groups (0–14, 15–24, and 25–39 years). As described above, analyses were also conducted using two age groups (0–17 years and 18–39 years).

Age-standardised incidence rates with 95% confidence intervals (CIs) were calculated per 100,000 person-years in 10-year periods from 1990 to 2019, using the Eurostat European Standard Population (reweighted to include only 0–40-year-olds).

Five-year relative survival rates with 95% CIs were calculated for the main strata and each of the following sub-strata: period of diagnosis (1990–1999, 2000–2009, 2010–2020), sex, site of primary tumour (upper or lower extremity and axial skeleton), primary tumour size at diagnosis (largest diameter ≤ 8 cm, > 8 cm, or not recorded), metastatic status at diagnosis (localised, metastatic, or undetermined/unknown), and initial treatment regimen (using different treatment groupings for the two cancer types).

Five-year survival rates were calculated using the cohort method, meaning that we included only patients with at least 5 years of potential observation time (i.e. diagnosed 5 years or more before the cut-off date of our vital status data). Patients who immigrated or were registered as disappeared within 5 years of diagnosis were excluded. Relative survival rates were calculated using the Ederer II method to estimate excess mortality compared with the background population [26]. Age- and calendar-year-specific mortality in the background population was calculated using the Danish Civil Registration System [19].

As a secondary analysis, we used logistic regression to examine the univariate associations of specific patient characteristics with the risk of death within 5 years. As we did not expect to have sufficient data for multivariable models, these analyses were intended only as exploratory.

The likelihood of progression/relapse and/or death within 5 years of diagnosis was examined for patients diagnosed in 2002–2023 (see description of available data above) using a multi-state model with the Aalen-Johansen estimator [27]. As this was a smaller dataset, analyses were conducted in two age groups (0–17 and 18–39 years).

Data management and analyses were performed in RStudio (version 1.4.1717) in a computing environment provided by Statistics Denmark. In accordance with Statistics Denmark’s requirements, we were not permitted to analyse or report on any strata with fewer than five individuals or fewer than five events (deaths). For this reason, the exact number of patients and/or the relative survival rate is not shown for some strata.

## Results

Between 1990 and 2024 in Denmark, 578 CAYA were diagnosed with either osteosarcoma (*n* = 336) or Ewing sarcoma of bone (*n* = 242). Clinical characteristics of patients are shown by sarcoma type and age group in Tables 1 and 2. (For results by two age groups, see Supplementary Materials 2). Any differences mentioned below are observed trends and are not necessarily statistically significant.

**Table 1:** Characteristics of patients diagnosed with osteosarcoma before the age of 40, in the years 1990–2024 in Denmark.

Patient age group	0-14 years		15-24 years		25-39 years	
	*n*	(%)*	*n*	(%)*	*n*	(%)*
Overall	121		137		78	
Period of diagnosis						
1990–1999	32	(26.4)	45	(32.8)	23	(29.5)
2000–2009	35	(28.9)	35	(25.5)	33	(42.3)
2010–2019	38	(31.4)	38	(27.7)	17	(21.8)
2020–2024	16	(13.2)	19	(13.9)	5	(6.4)
Sex						
Female	55	(45.5)	55	(40.1)	46	(59.0)
Male	66	(54.5)	82	(59.9)	32	(41.0)
Primary tumour site						
Upper-extremity bone	20	(16.5)	24	(17.5)	9	(11.5)
Lower-extremity bone	85–88	(70.2–72.7)	88–91	(64.2–66.4)	43	(55.1)
Pelvis	7	(5.8)	9	(6.6)	7	(9.0)
Non-pelvic axial bone	5	(4.1)	12	(8.8)	14	(17.9)
Unspecified bone	< 5	(< 4.1)	< 5	(< 3.6)	5	(6.4)
Largest tumour diameter **						
≤ 8 cm	52	(43.0) [61.9]	44	(32.1) [52.4]	21	(26.9) [51.2]
> 8 cm	32	(26.4) [38.1]	40	(29.2) [47.6]	20	(25.6) [48.8]
Not recorded	37	(30.6)	53	(38.7)	37	(47.4)
Metastatic stage at diagnosis						
Localised	90–93	(74.4–76.9)	101	(73.7)	60	(76.9)
Metastatic	27	(22.3)	29	(21.2)	12	(15.4)
Undetermined/not recorded	< 5	(< 4.1)	7	(5.1)	6	(7.7)
Primary treatment modalities						
Surgery + chemotherapy	71–74	(58.7–61.2)	59	(43.1)	26	(33.3)
Surgery only	5	(4.1)	6	(4.4)	6	(7.7)
Other	< 5	(< 4.1)	13	(9.5)	7	(9.0)
Not recorded	41	(33.9)	59	(43.1)	39	(50.0)

Some numbers given as ‘< 5’ to comply with requirements of Statistics Denmark. To prevent imputation of the exact number, the size of the largest group under the same category is given as an interval.

* : Percentages may not sum to 100.0 for each category because of rounding.

** : Relative distribution of tumours with reported size is shown in square brackets.

**Table 2:** Characteristics of patients diagnosed with Ewing sarcoma of bone before the age of 40, in the years 1990–2024 in Denmark.

Patient age group	0-14 years		15-24 years		25-39 years	
	*n*	(%)*	*n*	(%)*	*n*	(%)*
Overall	98		97		47	
Period of diagnosis						
1990–1999	18	(18.4)	11	(11.3)	11	(23.4)
2000–2009	40	(40.8)	34	(35.1)	12	(25.5)
2010–2019	34	(34.7)	40	(41.2)	17	(36.2)
2020–2024	6	(6.1)	12	(12.4)	7	(14.9)
Sex						
Female	47	(48.0)	35	(36.1)	20	(42.6)
Male	51	(52.0)	62	(63.9)	27	(57.4)
Primary tumour site						
Upper-extremity bone	12	(12.2)	11	(11.3)	6	(12.8)
Lower-extremity bone	27	(27.6)	37–40	(38.1–41.2)	15–18	(31.9–38.3)
Pelvis	20	(20.4)	25	(25.8)	6	(12.8)
Non-pelvic axial bone	39	(39.8)	20	(20.6)	16	(34.0)
Unspecified bone	0	-	< 5	(< 5.2)	< 5	(< 10.6)
Largest tumour diameter **						
≤ 8 cm	48	(49.0) [66.7]	29	(29.9) [38.2]	10	(21.3) [37.0]
> 8 cm	24	(24.5) [33.3]	47	(48.5) [61.8]	17	(36.2) [63.0]
Not recorded	26	(26.5)	21	(21.6)	20	(42.6)
Metastatic stage at diagnosis						
Localised	68	(69.4)	61–64	(62.9–66.0)	24–27	(51.1–57.4)
Metastatic	30	(30.6)	32	(33.0)	19	(40.4)
Undetermined/not recorded	0	-	< 5	(< 5.2)	< 5	(< 10.6)
Primary treatment modalities						
Surgery + chemotherapy	23	(23.5)	30	(30.9)	12	(25.5)
Surgery + chemotherapy + radiation	32	(32.7)	25	(25.8)	10	(21.3)
Other	6	(6.1)	18	(18.6)	5	(10.6)
Not recorded	37	(37.8)	24	(24.7)	20	(42.6)

Some numbers are reported as ‘< 5’ to comply with Statistics Denmark requirements. To prevent imputation of the exact number, the size of the largest group under the same category is given as an interval.

* : Percentages may not sum to 100.0 for each category because of rounding.

** : Relative distribution of tumours with reported size is shown in square brackets.

For both sarcoma types, sex distribution varied by age group. For osteosarcoma, males predominated among patients aged 0–14 and 15–24 years, whereas females predominated among those aged 25–39 years. For Ewing sarcoma of bone, there was no sex difference in the youngest age group, but males predominated in the two older groups.

The distribution of primary tumour sites also varied by age group. For osteosarcoma, lower-extremity tumours were most prevalent across all ages, but in the oldest patients, the proportion of extremity tumours decreased relative to axial tumours. In contrast, Ewing sarcomas showed a more heterogeneous anatomical distribution, with axial tumours occurring more frequently in the youngest patients than in the two older age groups.

Information on tumour size was available only for patients diagnosed after 2001. Among patients with a reported tumour size (see square brackets in Tables 1 and 2), the proportion with large primary tumours increased with age in both sarcoma types, most markedly in Ewing sarcoma.

For both sarcoma types, the distribution of localised versus metastatic disease at diagnosis appeared similar across the 0–14 and 15–24 age groups. The 25–39 age group had a slightly lower prevalence of metastatic osteosarcoma and a higher prevalence of metastatic Ewing sarcoma than the younger age groups.

Information on treatment was available only for patients diagnosed after 2001. For osteosarcoma, surgery combined with chemotherapy was by far the most common treatment. The proportion of patients receiving other treatments was slightly higher in the older age groups than in the youngest. For Ewing sarcoma, treatment regimens of either surgery plus chemotherapy or surgery plus chemotherapy plus radiation therapy were equally common in the study population, with some variation in their relative distribution across the age groups.

Age-standardised incidence rates are shown in Table 3. Apart from a lower rate for Ewing sarcoma in 1990–1999, incidence rates appear stable across the three decades, and similar for the two types of bone sarcoma.

**Table 3:** Age-standardised incidence rates of osteosarcoma and Ewing sarcoma of bone among 0–39-year-olds in Denmark in 1990–2019.

Period	Osteosarcoma		Ewing sarcoma of bone	
	ASIR*	(95% CI)	ASIR*	(95% CI)
1990–1999	0.36	(0.29–0.44)	0.14	(0.10–0.20)
2000–2009	0.37	(0.31-0.45)	0.31	(0.25–0.39)
2010–2019	0.33	(0.27-0.40)	0.32	(0.26–0.39)

ASIR: age-standardised incidence rate; CI: confidence interval.

* : Per 100,000 life-years.

### Five-year relative survival from osteosarcoma (1990–2020)

Table 4 shows the 5-year relative survival by age group for patients with osteosarcoma.

**Table 4:** Relative 5-year survival of patients diagnosed with osteosarcoma before age 40, in the years 1990–2020 in Denmark.

Patient age group	0-14 years			15-24 years			25-39 years		
	*n* at risk	5–year RS	(95% CI)	*n* at risk	5–year RS	(95% CI)	*n* at risk	5–year RS	(95% CI)
Overall	106	0.60	(0.50–0.69)	118	0.56	(0.46–0.64)	74	0.59	(0.47–0.70)
Period of diagnosis									
1990–1999	32	0.50	(0.32–0.66)	45	0.53	(0.37–0.66)	23	0.61	(0.38–0.77)
2000–2009	35	0.60	(0.42–0.74)	34	0.58	(0.40–0.73)	33	0.64	(0.45–0.78)
2010–2020	39	0.68	(0.51–0.81)	39	0.57	(0.40–0.71)	18	0.50	(0.26–0.70)
Sex									
Female	49	0.55	(0.40–0.67)	41	0.71	(0.55–0.82)	45	0.60	(0.45–0.73)
Male	57	0.65	(0.51–0.76)	71	0.46	(0.35–0.57)	29	0.58	(0.39–0.74)
Primary tumour site									
Upper-extremity bone	17	0.39	(0.17–0.60)	18	0.57	(0.32–0.75)	9	0.35	(0.10–0.62)
Lower-extremity bone	74–77	0.64	(0.53–0.74)	78–81	0.60	(0.48–0.70)	40	0.73	(0.56–0.84)
Axial bone*	11	0.72	(0.35–0.90)	18	0.50	(0.26–0.70)	20	0.47	(0.25–0.66)
Unspecified bone	< 5	–	–	< 5	–	–	5	–	–
Largest tumour diameter									
≤ 8 cm	45	0.75	(0.59–0.85)	32	0.69	(0.50–0.82)	20	0.74	(0.48–0.88)
> 8 cm	24	0.45	(0.25–0.63)	34	0.47	(0.29–0.62)	17	0.52	(0.27–0.72)
Not recorded	37	0.52	(0.35–0.67)	52	0.54	(0.39–0.66)	37	0.55	(0.39–0.69)
Metastatic stage at diagnosis									
Localised	78–81	0.72	(0.60–0.80)	83	0.67	(0.55–0.76)	56	0.69	(0.55–0.79)
Metastatic	24	0.27	(0.12–0.44)	28	0.31	(0.15–0.48)	12	0.35	(0.11–0.61)
Undetermined/not recorded	< 5	–	–	7	–	–	6	–	–
Primary treatment modalities									
Surgery + chemotherapy	60	0.66	(0.52–0.76)	45	0.69	(0.53–0.80)	22	0.64	(0.41–0.80)
Other	5	–	–	17	0.43	(0.20–0.65)	13	0.69	(0.37–0.87)
Not recorded	41	0.49	(0.33–0.63)	56	0.49	(0.36–0.62)	39	0.54	(0.37–0.68)

RS: relative survival; CI: confidence interval.

Some numbers are reported as ‘< 5’ to comply with Statistics Denmark requirements. To prevent imputation of the exact number, the size of the largest group under the same category is given as an interval.

* : All axial tumours combined to comply with requirement for minimum number of probands and events.

Over the study period, 5-year relative survival increased among 0–14-year-olds (from 0.50 to 0.68), remained close to stationary among 15–24-year-olds (from 0.53 to 0.57), and decreased among 25–39-year-olds (from 0.61 to 0.50).

Sex-dependent differences in relative survival were observed in 0–14-year-olds (F: 0.55 vs. M: 0.65) and 15–24-year-olds (F: 0.71 vs. M: 0.46), with only a minor difference in the oldest patients (F: 0.60 vs. M: 0.58).

Relative survival from upper-extremity tumours was markedly lower than from lower-extremity tumours in the youngest and oldest age groups. For patients with lower-extremity tumours (the most common site), relative survival rates were similar in the two younger age groups (0.64 and 0.60), and higher in the oldest age group (0.73). Relative survival from axial tumours was similar in the middle and oldest age groups (0.50 and 0.47), and highest in the youngest patients (0.72).

Having a large primary tumour (largest diameter > 8 cm) or metastatic disease at diagnosis reduced relative survival (by 22 to 45 percentage points) in all three age groups. There were only minor differences in relative survival across age groups stratified by tumour stage or metastatic status.

Relative survival after primary treatment with surgery and chemotherapy was similar in all three age groups. Relative survival after other primary treatment regimens was better in 25–39-year-olds than in 15–24-year-olds.

### Five-year relative survival from Ewing sarcoma of bone (1990–2020)

Table 5 shows 5-year relative survival by age group for patients with Ewing sarcoma of bone.

**Table 5:** Relative 5-year survival of patients diagnosed with Ewing sarcoma of bone before age 40, in the years 1990–2020 in Denmark.

Patient age group	0-14 years			15-24 years			25-39 years		
	*n* at risk	5–year RS	(95% CI)	*n* at risk	5–year RS	(95% CI)	*n* at risk	5–year RS	(95% CI)
Overall	92	0.64	(0.53–0.72)	84	0.59	(0.47–0.68)	42	0.54	(0.37–0.68)
Period of diagnosis									
1990–1999	17	0.50	(0.26–0.70)	10	0.45	(0.15–0.72)	11	0.42	(0.14–0.68)
2000–2009	40	0.64	(0.47–0.77)	33	0.51	(0.33–0.66)	12	0.83	(0.46–0.96)
2010–2020	35	0.70	(0.52–0.83)	41	0.68	(0.52–0.80)	19	0.42	(0.20–0.63)
Sex									
Female	45	0.67	(0.51–0.78)	29	0.65	(0.45–0.79)	18	0.56	(0.30–0.76)
Male	47	0.60	(0.42–0.74)	55	0.55	(0.41–0.67)	24	0.52	(0.31–0.70)
Primary tumour site									
Upper-extremity bone	12	0.92	(0.54–0.99)	9	0.77	(0.35–0.94)	6	0.65	(0.18–0.90)
Lower-extremity bone	26	0.72	(0.51–0.86)	30–33	0.70	(0.50–0.83)	15	0.45	(0.18–0.68)
Pelvis	19	0.41	(0.20–0.61)	22	0.51	(0.28–0.70)	5	–	–
Non-pelvic axial bone	35	0.60	(0.42–0.74)	19	0.50	(0.28–0.69)	12–15	0.68	(0.39–0.85)
Unspecified bone	0	–	–	< 5	–	–	< 5	–	–
Largest tumour diameter									
≤ 8 cm	46	0.73	(0.58–0.84)	24	0.75	(0.53–0.88)	10	0.66	(0.27–0.88)
> 8 cm	21	0.54	(0.31–0.73)	40	0.54	(0.37–0.68)	13	0.44	(0.17–0.67)
Not recorded	25	0.53	(0.33–0.70)	20	0.48	(0.25–0.67)	19	0.55	(0.30–0.74)
Metastatic stage at diagnosis									
Localised	65	0.75	(0.63–0.84)	56–59	0.73	(0.60–0.83)	19–22	0.69	(0.44–0.85)
Metastatic	27	0.35	(0.18–0.52)	24	0.33	(0.16–0.52)	19	0.32	(0.14–0.53)
Undetermined/not recorded	0	–	–	< 5	–	–	< 5	–	–
Primary treatment modalities									
Surgery + chemotherapy	22	0.91	(0.67–0.98)	24	0.75	(0.53–0.88)	11	0.71	(0.34–0.90)
Surgery + chemo + radiation	30	0.59	(0.40–0.74)	22	0.73	(0.50–0.87)	9	0.41	(0.12–0.69)
Other	< 5	–	–	15	0.37	(0.15–0.60)	< 5	–	–
Not recorded	36–39	0.50	(0.34–0.65)	23	0.41	(0.21–0.59)	18–21	0.55	(0.30–0.74)

RS: relative survival; CI: confidence interval.

Some numbers are reported as ‘< 5’ to comply with Statistics Denmark requirements. To prevent imputation of the exact number, the size of the largest group under the same category is given as an interval.

Over the study period, 5-year relative survival increased by approximately 20 percentage points in the two youngest age groups, reaching 0.70 and 0.68 in 2010–2018. In the oldest age group, relative survival fluctuated but showed no overall trend towards improvement, with identical rates of 0.42 in the first and last calendar periods.

Females had better relative survival than males across all three age groups, with the sex-dependent difference being largest in 15–24-year-olds.

Tumours in the extremities carried markedly higher relative survival rates than axial tumours in the two younger age groups, with upper-extremity tumours being favourable to lower-extremity ones. The oldest patients had markedly lower relative survival rates for extremity tumours, but higher relative survival rates for non-pelvic axial tumours, when compared to the younger age groups.

Having a large primary tumour (largest diameter > 8 cm) or metastatic disease at diagnosis reduced relative survival (by 19 to 40 percentage points) across all three age groups. The two youngest age groups had near-identical relative survival stratified by tumour stage or metastatic status. In the oldest age group, relative survival from large primary tumours was lower by 10 percentage points, while relative survival from smaller tumours and from localised and metastatic disease were only slightly lower than in the two youngest age groups.

Relative survival after primary treatment with surgery and chemotherapy was markedly higher in 0–14-year-olds (0.91) than in the two older age groups (0.75 and 0.71). Relative survival after primary treatment with surgery, chemotherapy, and radiation therapy was highest among 15–24-year-olds (0.73) and lowest among 25–39-year-olds (0.41).

### Univariate risk estimates

Results of the exploratory univariate logistic regressions are shown in Supplemental Materials 3. For both sarcoma types, the risk of death within 5 years of diagnosis was significantly higher in patients with large primary tumours, metastatic disease or primary tumours in the pelvis (compared with lower-extremity tumours). For osteosarcoma, the risk of death within 5 years was also significantly higher for upper-extremity tumours (compared with lower-extremity tumours). For Ewing sarcoma, the risk of death within 5 years was significantly higher in 18–39-year-olds than in 0–17-year-olds.

### Likelihood of progression/relapse and death (2002–2023)

**Figure 1:**
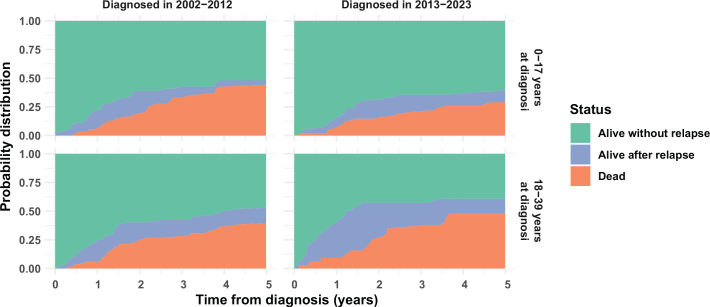
Stacked probabilities of relapse/progression and death within 5 years from diagnosis in patients diagnosed with osteosarcoma in 2002–2023 in Denmark

**Figure 2:**
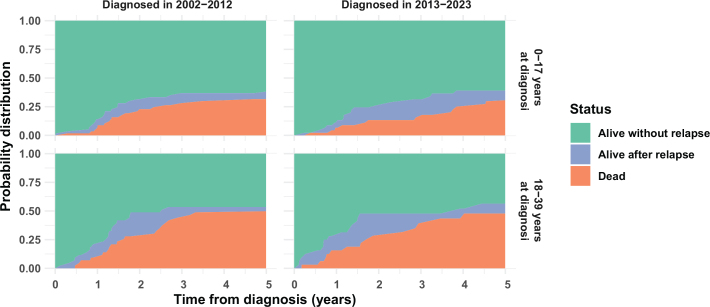
Stacked probabilities of relapse/progression and death within 5 years from diagnosis in patients diagnosed with Ewing sarcoma of bone in 2002 2023 in Denmark.

Figures 1 and 2 show stacked probabilities (given by the Aalen-Johansen estimator) of being alive without relapse/progression, alive with relapse/progression, or dead at a given time in the 5 years following diagnosis for patients diagnosed in 2002–2023. Green areas correspond to event-free survival. The combined green and blue areas correspond to overall survival.

For patients diagnosed with either sarcoma type in 2013–2023, those aged 0–17 years at diagnosis were more likely to survive for 5 years (both overall survival and event-free survival) than those aged 18–39 years at diagnosis.

Patients diagnosed in 2013–2023 appeared to survive for longer after relapse/progression than those diagnosed in 2002–2012. This was most evident among young adults with osteosarcoma, but the effect was observed across both sarcoma types and age groups.

## Discussion and conclusion

### Main findings and perspectives

In this study, we used a unique dataset that combined population-wide register data with clinical data from hospital records to demonstrate disparities in 5-year relative survival among clinically relevant subgroups of CAYA diagnosed with osteosarcoma or Ewing sarcoma of bone.

We find that 5-year relative survival from both osteosarcoma and Ewing sarcoma of bone has improved for patients aged 0–24 years over the past three decades. In contrast, relative survival for patients aged 25–39 years appears to have stagnated over the same period and may even have declined for osteosarcoma. This widening disparity between age groups, likely multifactorial, has also been observed in other bone cancer cohorts:

Firstly, a multinational European cohort study of CAYA patients diagnosed with sarcomas between 2006 and 2013 reported a 5-year RS, similar to our findings for both osteosarcoma and Ewing sarcoma of bone (although that study combined all patients aged 15–39 into a single age group) [14].

Secondly, a recent population-based cohort study of CAYA patients diagnosed with Ewing sarcoma in the Netherlands between 1990 and 2018 found comparable survival disparities between two age groups (0–17 and 18–39 years) and identified age-dependent differences in the anatomical distribution of primary tumours and disease stage as possible mediators [15].

Our findings corroborate these findings. For osteosarcoma, extremity tumours were more common in the two younger age groups, whereas axial tumours were both more common and associated with poorer survival in patients aged 25–39 years. Although there were no obvious age-dependent differences in relative survival rates from osteosarcoma when stratified by tumour size, metastatic state or primary treatment, we did observe an age-dependent difference in the proportion of patients with large tumours (49% in 25–39-year-olds compared with 38% in 0–14-year-olds). We also found that a smaller proportion of 25–39-year-olds underwent primary surgery. These three findings suggest that older patients may be more likely to have inoperable tumours at diagnosis, reducing the likelihood of curative treatment.

For Ewing sarcoma of bone, several observed factors could contribute to the age group difference in relative survival. Firstly, 25–39-year-olds had a similar proportion of extremity tumours as the younger age groups, but markedly lower relative survival from these tumours. This could be mitigated by a higher proportion of, and higher relative survival from, tumours in non-pelvic axial bones within the same age group. Secondly, relative survival from large primary tumours was 10 percentage points lower in the oldest age group than in the two youngest groups. Relative survival from smaller tumours and from localised and metastatic disease was also slightly lower among 25–39-year-olds than among younger patients. Finally, the oldest patients had the lowest relative survival when stratified by the two main primary treatment regimens.

Altogether, our findings suggest that several interconnected factors contribute to lower relative survival in the 25–39-year age group for both sarcoma types, including age-dependent variations in anatomical distribution and disease stage at diagnosis, which may limit the options for curative treatment. This may, in turn, increase the risk of relapse in older patients, as supported by our findings shown in Figures 1 and 2, and decrease survival rates.

These survival disparities may also be mediated by age-dependent differences in patient- and/or tumour response to treatment, although this study could not examine such associations. Our group is currently preparing a study of treatment intensity and toxicities across different age groups in CAYA with bone sarcomas.

Sex-dependent survival differences, of varying magnitude, favoured females in five of the six sarcoma-type and age-group strata. The largest discrepancies were observed in 15–24-year-olds for both sarcoma types. For both OS and ES, sex is less well-documented as an independent prognostic factor for survival than age. Recent systematic reviews of prognostic factors in osteosarcoma [13] and Ewing sarcoma [23] found that, in each disease, two studies reported significantly lower survival in males, whereas most studies did not identify sex as an independent prognostic factor.

Given that poorer survival in females has not previously been reported, the observed lower 5-year relative survival in females compared to males among osteosarcoma patients aged 0–14 years was somewhat surprising. We conducted a post hoc comparison of the case mix between females and males aged 0–14 years with osteosarcoma and found only minor differences in distributions of clinical presentation and chosen treatment regimen (data not shown).

Any association between sex and survival is not obvious from this or previous studies. Possible mediators of sex-dependent survival differences could be either fixed, biological effects or modifiable, behavioural ones. Sex-dependent health-behavioural differences could affect trajectories within the healthcare system [28, 29]. Biological effects could include differences in disease presentation and/or treatment tolerance [30]. Sex-dependent differences in tumour genomics and microenvironment have also been suggested [31], and one future path to reduce survival inequity could be an increased use of sequencing to find actionable mutations for individualised treatment [32].

Altogether, our findings suggest that several patient-, disease-, and treatment-related factors influence 5-year survival, and that these factors interact in ways that are not entirely understood. Interestingly, a long-term follow-up analysis of the Cooperative Osteosarcoma Study Group (COSS) cohort also suggests that determinants of survival beyond 5 years may increasingly relate to factors occurring along the treatment trajectory rather than tumour or patient characteristics at diagnosis, further underscoring the importance of recording detailed clinical data in long-term follow-up [33].

### Strengths and limitations

Danish national registers encompass the entire population, with individuals uniquely identifiable by a personal identification number, allowing data to be merged across registers and healthcare records. All cancers diagnosed in Denmark are automatically registered in the Danish Cancer Register, enabling us to identify and include all relevant patients within the study period, with additional validation of all patients diagnosed after 2002 using the Danish National Pathology Bank [19]. The addition of clinical data from electronic hospital records resulted in a unique dataset. Our overall findings that increasing age, non-extremity tumours, larger primary tumours, and metastatic disease are linked to lower relative survival align with existing knowledge and support the external validity of the study, including results from large registry-based clinical cohorts such as those reported by the COSS [34].

The study’s primary limitation was the size of the study population. Despite nationwide coverage over several decades, the rarity of bone sarcomas limited the inferential analyses. Even simple stratification by selected, clinically relevant characteristics resulted in wide CIs. With this caveat, we still believe that our results demonstrate clinically relevant trends as discussed above. Further disentangling the effects of specific clinical characteristics on the risk of death will require multivariable inferential models and, consequently, larger data sets. One solution is to pool data from countries with similar healthcare systems, for example, within the already established cancer network for the Nordic countries, NordCan.

Another relevant limitation is the risk of misclassifications in the data, as the diagnostic methods and classification of bone sarcomas are continually evolving. For instance, changes in histopathological methods may have caused inconsistent classification of Ewing sarcoma throughout the study period (see Table 3), and advances in imaging technology (e.g. the adoption of PET/CT) may have influenced the observed prevalence of metastatic disease at diagnosis. Nonetheless, these developmental changes are likely similar in other high-income countries, and thus our results should still be comparable to those of other studies.

Classifying tumour size using a unidimensional measure is less accurate than using volumetric definitions, but we lacked sufficient data to reliably determine tumour volume. Since this was a descriptive study focused on comparing age groups rather than developing a prognostic model, we considered this limitation acceptable. The definition employed in this study has precedence in existing literature.

In Denmark, we have historically used contemporary international protocols as clinical guidelines for the treatment of CAYA with sarcoma. Still, the number of patients included as trial participants has generally been very low, often because the relevant protocols have not been open for inclusion. As a result, no meaningful analyses comparing trial patients with non-trial patients could be conducted. Low participation rates in clinical trials hinder progress in treatment development and in identifying better treatment strategies for future patients [35].

### Conclusion

In Denmark, over the past three decades, 5-year relative survival rates for both osteosarcoma and Ewing sarcoma of bone have improved for patients aged 0–24 years but have plateaued for those aged 25–39 years. Several interconnected factors seem to contribute to this age-dependent survival disparity, including variations in tumour location and size, as well as differences in initial treatment regimens, possibly increasing the risk of relapse. Future efforts to further understand survival inequities could include studies of patient trajectories within the healthcare system and studies of tumour genomics and microenvironments.

## Supplementary Material



## Data Availability

Due to the EU General Data Protection Regulation and Danish national data regulations, the register data used cannot be made available by the authors. Analysis scripts can be made available upon reasonable request.
