# Supplementary material for: Increasing survival disparity between children, adolescents, and young adults with osteosarcoma or Ewing sarcoma of bone from 1990 to 2024: a population-based cohort study
Source: Acta Oncol. 2026 Apr 22;65:45405. doi: 10.2340/ao.v65.45405 (PMC13112519; doi:10.2340/ao.v65.45405)
Supplement: Supplementary file 1 [file AO-65-45405-s1.pdf]

Supplementary material has been published as submitted. It has not been copyedited, or typeset by Acta Oncologica

Supplementary Materials 1.

Table A. ICD-O-3 codes used to define the study population.

	ICD-O-3 codes	Description	n in study population
**Morphology codes**			
Osteosarcoma (OS)	9180/3<br>9181/3<br>9182/3<br>9183/3<br>9184/3<br>9185/3<br>9186/3<br>9187/3<br>9192/3<br>9193/3<br>9194/3<br>9195/3	OS, NOS<br>Chondroblastic OS<br>Fibroblastic OS<br>Telangiectatic OS<br>OS in Paget's Disease<br>Small cell OS<br>Central/medullary OS<br>Intraosseous low grade OS<br>Parosteal OS<br>Periosteal OS<br>High grade surface OS<br>Intracortical OS	272<br>32<br>6<br>7<br>0<br>0<br>0<br><5<br>15<br><5<br>0<br>0
Ewing sarcoma	9260/3<br>9364/3<br>9365/3	Ewing sarcoma<br>pPNET*<br>Askin tumor	235<br><5<br><5
**Topography codes**			
Upper extremity bone	C40.0<br>C40.1	Long –<br>Short bones of upper limb, scapula and associated joints	69<br>13
Lower extremity bone	C40.2<br>C40.3	Long –<br>Short bones of upper limb, scapula and associated joints	280<br>20
Pelvic bone	C41.4	Pelvic bones, sacrum, and coccyx	74
Non-pelvic axial bone	C41.0<br>C41.1<br>C41.2<br>C41.3	Bones of skull and face<br>Mandible<br>Vertebral column<br>Rib, sternum, clavicle, and associated joints	29<br>8<br>22<br>47
Unspecified bone	C40.8<br>C40.9<br>C41.8<br>C41.9	Overlapping lesion of bones, joints and articular cartilage of limbs<br>Bone of limbs, NOS<br>Overlapping lesion of bones, joints and articular cartilage<br>Bone, NOS	0<br>5<br><5<br>7

*: peripheral primitive neuroectodermal tumors

**: undifferentiated small round cell tumors

All patients included in the study had both a morphology and a topography code matching one of the above codes.

Supplementary Materials 2

Table A. Characteristics of patients diagnosed with **osteosarcoma** before age 40, in the years **1990-2024** in Denmark.

	Age 0-17		Age 18-39	
	n	(%)*	n	(%)*
Overall	177		159	
Period of diagnosis				
1990-1999	49	(27.7)	51	(32.1)
2000-2009	47	(26.6)	56	(35.2)
2010-2019	56	(31.6)	38	(23.9)
2020-2024	25	(14.1)	14	(8.8)
Sex				
Female	81	(45.8)	75	(47.2)
Male	96	(54.2)	84	(52.8)
Primary tumor site				
Upper extremity bone	28	(15.8)	25	(15.7)
Lower extremity bone	128–131	(72.3–74.0)	90	(56.6)
Pelvis	10	(5.6)	13	(8.2)
Non-pelvic axial bone	7	(4.0)	24	(15.1)
Unspecified bone	<5	(<2.8)	7	(4.4)
Largest tumor diameter **				
≤ 8 cm	66	(37.3) [54.5]	51	(32.1) [58.0]
> 8 cm	55	(31.1) [45.5]	37	(23.3) [42.0]
Not recorded	56	(31.6)	71	(44.7)
Metastatic stage at diagnosis				
Localized	131	(74.0)	121	(76.1)
Metastatic	41	(23.2)	27	(17.0)
Undetermined/not recorded	5	(2.8)	11	(6.9)
Primary treatment modalities				
Surgery and chemotherapy	102–105	(57.6–59.3)	56	(35.2)
Surgery only	7	(4.0)	11	(6.9)
Other	<5	(<2.8)	17	(10.7)
Not recorded	64	(36.2)	75	(47.2)

Some numbers are given as “<5” to comply with requirement of Statistics Denmark. To prevent imputation of the exact number, the size of the largest group under the same category is given as an interval.

*: Percentages may not sum to 100.0 for each category because of rounding.

**: Relative distribution of tumors with reported size is shown in square brackets.

Supplementary Materials 2.

Table B. Characteristics of patients diagnosed with **Ewing sarcoma of bone** before age 40, in the years **1990-2024** in Denmark.

	**Age 0-17**		Age 18-39	
	n	(%)*	n	(%)*
Overall	138		104	
Period of diagnosis				
1990-1999	22	(15.9)	18	(17.3)
2000-2009	49	(35.5)	37	(35.6)
2010-2019	55	(39.9)	36	(34.6)
2020-2024	12	(8.7)	13	(12.5)
Sex				
Female	62	(44.9)	40	(38.5)
Male	76	(55.1)	64	(61.5)
Primary tumor site				
Upper extremity bone	16	(11.6)	13	(12.5)
Lower extremity bone	44	(31.9)	38–41	(36.5–39.4)
Pelvis	30	(21.7)	21	(20.2)
Non-pelvic axial bone	44–47	(31.9–34.1)	28	(26.9)
Unspecified bone	<5	(<3.6)	<5	(<4.8)
Largest tumor diameter **				
≤ 8 cm	62	(44.9) [59.0]	25	(24.0)
> 8 cm	43	(31.2) [41.0]	45	(43.3)
Not recorded	33	(23.9)	34	(32.7)
Metastatic stage at diagnosis				
Localized	96	(69.6)	59	(56.7)
Metastatic	42	(30.4)	39	(37.5)
Undetermined/not recorded	0	-	6	(5.8)
Primary treatment modalities				
Surg + chemo	36	(26.1)	29	(27.9)
Surg + chemo + rad	42	(30.4)	25	(24.0)
Other	15	(10.9)	14	(13.5)
Not recorded	45	(32.6)	36	(34.6)

Some numbers are given as "<5" to comply with requirement of Statistics Denmark. To prevent imputation of the exact number, the size of the largest group under the same category is given as an interval.

*: Percentages may not sum to 100.0 for each category because of rounding.

**: Relative distribution of tumors with reported size is shown in square brackets.

Supplementary Materials 2.

Table C. Relative **5-year** survival of patients diagnosed with **osteosarcoma** before age 40, in the years **1990-2020** in Denmark.

	Age 0-17			Age 18-39		
	n at risk	5-year RS	(95% CI)	n at risk	5-year RS	(95% CI)
Overall	154	0.58	(0.50–0.66)	144	0.58	(0.50–0.66)
Period of diagnosis						
1990-1999	49	0.48	(0.34–0.61)	51	0.60	(0.45–0.72)
2000-2009	47	0.57	(0.42–0.70)	55	0.63	(0.49–0.75)
2010-2018	58	0.68	(0.54–0.78)	38	0.49	(0.32–0.63)
Sex						
Female	73	0.59	(0.47–0.69)	68	0.65	(0.52–0.75)
Male	81	0.58	(0.46–0.68)	76	0.52	(0.41–0.63)
Primary tumor site						
Upper extremity bone	24	0.41	(0.22–0.59)	20	0.51	(0.28–0.70)
Lower extremity bone	110–113	0.64	(0.54–0.72)	84	0.64	(0.53–0.74)
Pelvis	9	0.30	(0.07–0.59)	11	0.33	(0.10–0.59)
Non-pelvic axial bone	7	0.86	(0.34–0.98)	22	0.61	(0.38–0.77)
Unspecified bone	<5	–	–	7	–	–
Largest tumor diameter						
≤ 8 cm	54	0.76	(0.62–0.85)	43	0.69	(0.53–0.81)
> 8 cm	45	0.49	(0.34–0.63)	30	0.45	(0.27–0.61)
Not recorded	55	0.49	(0.35–0.61)	71	0.57	(0.45–0.68)
Metastatic stage at diagnosis						
Localized	112	0.70	(0.61–0.78)	106	0.68	(0.58–0.76)
Metastatic	37	0.28	(0.16–0.43)	27	0.32	(0.16–0.49)
Undetermined/not recorded	5	–	–	11	0.29	(0.08–0.54)
Primary treatment modalities						
Surgery and chemotherapy	81–84	0.67	(0.56–0.76)	45	0.65	(0.49–0.77)
Surgery only	6	1.00	(1.00–1.00)	10	0.90	(0.47–0.99)
Other	<5	–	–	16	0.34	(0.13–0.56)
Not recorded	63	0.46	(0.33–0.57)	73	0.55	(0.43–0.65)

Some numbers are given as “<5” to comply with requirement of Statistics Denmark. To prevent imputation of the exact number, the size of the largest group under the same category is given as an interval.

*: Percentages may not sum to 100.0 for each category because of rounding.

Supplementary Materials 2.

Table D. Relative **5-year** survival of patients diagnosed with **Ewing sarcoma of bone** before age 40, in the years **1990-2020** in Denmark.

	**Age 0-17**			**Age 18-39**		
	n at risk	5-year RS	(95% CI)	n at risk	5-year RS	(95% CI)
Overall	126	0.66	(0.57–0.74)	92	0.51	(0.40–0.60)
Period of diagnosis						
1990-1999	21	0.59	(0.36–0.76)	17	0.30	(0.11–0.52)
2000-2009	49	0.67	(0.51–0.78)	36	0.55	(0.37–0.69)
2010-2018	56	0.69	(0.55–0.80)	39	0.56	(0.39–0.70)
Sex						
Female	58	0.69	(0.56–0.79)	34	0.55	(0.37–0.70)
Male	68	0.64	(0.51–0.74)	58	0.48	(0.35–0.60)
Primary tumor site						
Upper extremity bone	15	0.86	(0.56–0.96)	12	0.75	(0.40–0.91)
Lower extremity bone	40	0.77	(0.61–0.87)	31-34	0.51	(0.32–0.67)
Pelvis	27	0.51	(0.32–0.68)	19	0.29	(0.11–0.50)
Non-pelvic axial bone	40–43	0.61	(0.45–0.74)	26	0.55	(0.34–0.71)
Unspecified bone	<5	–	–	<5	–	–
Largest tumor diameter						
≤ 8 cm	56	0.75	(0.61–0.84)	24	0.69	(0.46–0.84)
> 8 cm	38	0.57	(0.39–0.71)	36	0.47	(0.30–0.62)
Not recorded	32	0.63	(0.45–0.77)	32	0.41	(0.24–0.57)
Metastatic stage at diagnosis						
Localized	91	0.76	(0.66–0.84)	51	0.69	(0.54–0.80)
Metastatic	35	0.40	(0.24–0.55)	35	0.27	(0.15–0.42)
Undetermined/not recorded	0	–	–	6	–	–
Primary treatment modalities						
Surg + chemo	32	0.91	(0.73–0.97)	25	0.67	(0.45–0.82)
Surg + chemo + rad	38	0.58	(0.41–0.71)	23	0.69	(0.46–0.84)
Other	12	0.74	(0.39–0.91)	10	0.10	(0.01–0.30)
Not recorded	44	0.55	(0.39–0.68)	34	0.40	(0.23–0.55)

Some numbers are given as “<5” to comply with requirement of Statistics Denmark. To prevent imputation of the exact number, the size of the largest group under the same category is given as an interval.

*: Percentages may not sum to 100.0 for each category because of rounding.

Supplementary Materials 3.

Table A. Univariate estimates of hazard ratio for death within five years from diagnosis in patients diagnosed with **osteosarcoma** before age 40, in the years **1990-2020** in Denmark.

	HR	(95% CI)
Age group		
0-17	ref	
18-39	1.02	(0.74–1.42)
Period of diagnosis		
1990-1999	ref	
2000-2009	0.85	(0.57–1.28)
2010-2018	1.07	(0.72–1.60)
Sex		
Female	ref	
Male	1.27	(0.80–2.02)
Primary tumor site		
Lower extremity bone	ref	
Upper extremity bone	**2.26**	**(1.17–4.42)**
Pelvis	**3.50**	**(1.37–9.69)**
Non-pelvic axial bone	0.99	(0.34–2.07)
Largest tumor diameter		
≤ 8 cm	ref	
> 8 cm	**2.80**	**(1.49–5.36)**
Metastatic stage at diagnosis		
Localized	ref	
Metastatic	**4.30**	**(2.41–7.88)**

Supplementary Materials 3.

Table B. Univariate estimates of hazard ratio for death within five years from diagnosis in patients diagnosed with **Ewing sarcoma of bone** before age 40, in the years **1990-2020** in Denmark.

	HR	(95% CI)
Age group		
0-17	ref	
18-39	**1.54**	**(1.04–2.27)**
Period of diagnosis		
1990-1999	ref	
2000-2009	0.61	(0.36–1.05)
2010-2018	1.24	(0.77–2.01)
Sex		
Female	ref	
Male	1.41	(0.81–2.46)
Primary tumor site		
Lower extremity bone	ref	
Upper extremity bone	0.45	(0.14–1.27)
Pelvis	**2.38**	**(1.12–5.14)**
Non-pelvic axial bone	1.45	(0.73–2.89)
Largest tumor diameter		
≤ 8 cm	ref	
> 8 cm	**2.24**	**(1.15–4.43)**
Metastatic stage at diagnosis		
Localized	ref	
Metastatic	**4.63**	**(2.54-8.63)**
